# Overcoming the challenges of access to eye care through mHealth in Kenya

**Published:** 2022-06-07

**Authors:** Hillary Rono, Lily Kimetto

**Affiliations:** 1 Ophthalmologist: Kitale County and Referral Hospital, Kitale, Kenya and a consultant at Peek Vision, UK.; 2 Optometrist: Bethsaida Eye Centre, Kitale, Kenya

**An mHealth project in Kenya has improved eye health care in schools and in the community.**

**Figure:**
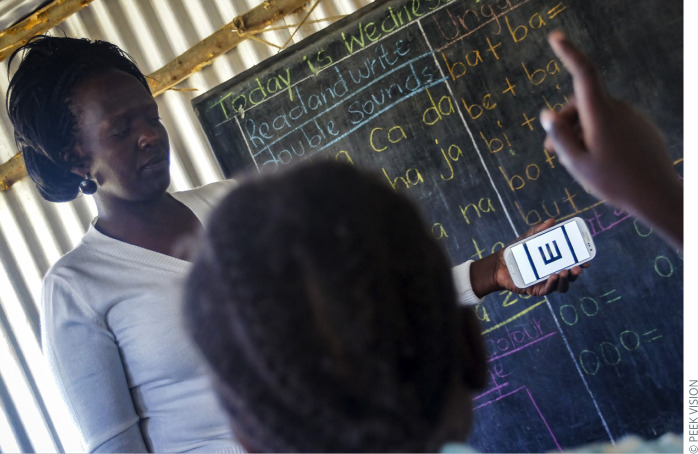
A teacher uses the Peek acuity app to screen children in her class for refractive error. **KENYA**

While cellphone access and mobile health (mHealth) technology continues to improve, there is a need for research to fully understand the potential of any mHealth solution before it can be more widely adopted and scaled up. Peek mHealth solutions were tested in two contexts in Kenya: a school eye health programme and a community eye health programme. The aims were to:

- create awareness and identify individuals with eye problems through eye screening
- improve access to eye care services
- increase the efficiency of existing eye care workers through guided task sharing.

The Peek mHealth solution uses Peek Acuity™ which is a validated smartphone app that accurately and reliably identifies people with vision impairment.^1^

## The school eye health programme

In the trial group, school teachers were trained to detect visual impairment in their students using the Peek Acuity smartphone app. Children who failed the vision test were sent home with specially designed referral cards that showed parents what their vision looked like and stated why they needed to get spectacles (or be seen by an eye specialist); the cards also stated where and when their appointments would take place. We also sent parents short message service (SMS) reminders about their children's appointments. The Peek system allowed us to track, in real time, who did and did not attend, so that we could send reminders again and make new appointments where needed. Compared to the control group, where children were tested with a card-based test and parents did not get SMS reminders (just standard referral letters) we found that the children in the trial group were more than twice as likely to attend the appointments (54% compared to 22%).^2^

## The community eye health programme

We ran a similar trial in the community, with the addition of a validated decision support algorithm^3^ that enabled community volunteers to decide whether to refer people to primary health facilities. If someone screened positive at the household level, they were automatically referred and sent SMS reminders about appointments. A cluster randomised trial showed that the combination of the algorithm and reminder messages nearly tripled primary care attendance by people with eye problems compared to standard approaches (1,429 per 10,000 residents in the Peek arm of the study, compared to 522 in the control arm), indicating the potential of this mobile health package to increase service uptake.^4^

A follow-up study of the participants identified and referred using this system in the community setting showed that, despite the SMS reminders, some participants did not attend further referrals from primary facilities to secondary facilities as they were not aware of the reason for referral. We learnt from this that we must include counselling and provide more patient information during the referral process.^5^ In other words, the team delivering primary eye care needed to further support the participants to understand their diagnosis and treatment options in order to improve their uptake of referrals.

## Adopting and scaling up

Adoption and scale-up of mHealth solutions involve identifying the strengths of various stakeholders and assigning responsibilities that maximise those strengths. In the scale-up of our school programme, for example, the stakeholders included local government, school leaders, the ministry of health, and the ministry of eduction. The ministry of health designed a policy guide, the local (county) government provided leadership and administrative support for the project, and the ministry of education offered insights into how policy and regulation were changing in the country and how our work needed to adapt. Open communication among stakeholders and constant review of the programme were also critical. Altogether, the scaled-up project resulted in a total of 168,820 children in Kenya being screened, of whom 6,200 were treated for eye problems.^6^

Evidence from the trials and the scale-up process was used to establish similar programmes in other countries; this also informed the inclusion of mHealth in Kenya's national eye care strategic plan as a means of supporting the provision of eye care services to the whole population.

## Challenges and considerations

We have identified some key emerging issues that must be considered when deploying mHealth solutions:

- the requirements of data protection laws in different jurisdictions
- how to manage the constant improvements in technology, requiring regular software or equipment updates and training of staff members
- the ability to be agile – i.e., to adapt and evolve how the programme or project is carried out, based on real-time data about what is, and is not, working
- how to remain in alignment with government priorities
- the availability of personnel to offer services.

**Figure:**
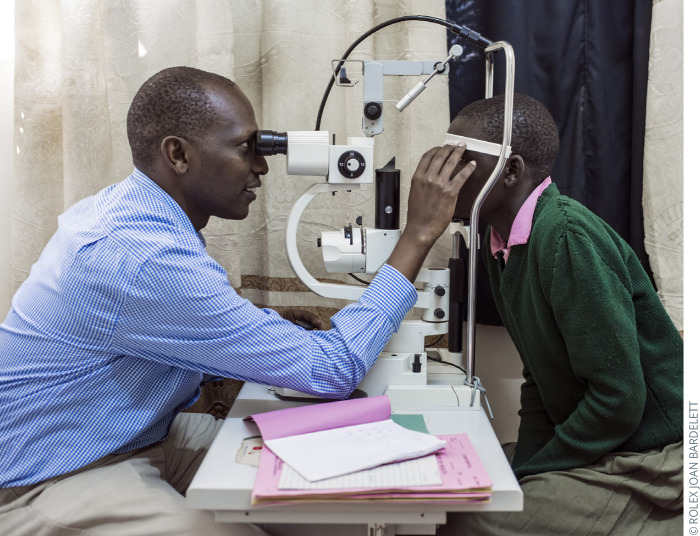
A child undergoes an eye examination at Kitale Eye Hospital after school screening using Peek technology identified potential vision problems. **KENYA**

Although mHealth solutions require eye care programme providers to continuously adapt programmes to meet the needs of the population, mHealth also supports and informs this process by allowing prompt analysis and sharing of programme data. This enables managers to monitor progress and make evidence-based decisions during programme implementation. mHealth solutions, therefore, allow for continuous improvement of programmes using locally generated data and make it possible for multiple stakeholders to be involved.

### Declaration of interests

*HR is a consultant at Peek Vision Ltd. UK. LK declares no competing interests.*
